# A grid-based infrastructure for ecological forecasting of rice land *Anopheles arabiensis *aquatic larval habitats

**DOI:** 10.1186/1475-2875-5-91

**Published:** 2006-10-24

**Authors:** Benjamin G Jacob, Ephantus J Muturi, Jose E Funes, Josephat I Shililu, John I Githure, Ibulaimu I Kakoma, Robert J Novak

**Affiliations:** 1 Illinois Natural History Survey, Center for Ecological Entomology, 1816 South Oak St. Champaign, IL 61820 Champaign IL 61820, USA; 2 Human Health Division, International Centre of Insect Physiology and Ecology (ICIPE), P.O. Box 30772, Nairobi, Kenya; 3 College of Veterinary Medicine, University of Illinois at Urbana-Champaign, 2001 South Lincoln Ave, Urbana IL 61802, USA

## Abstract

**Background:**

For remote identification of mosquito habitats the first step is often to construct a discrete tessellation of the region. In applications where complex geometries do not need to be represented such as urban habitats, regular orthogonal grids are constructed in GIS and overlaid on satellite images. However, rice land vector mosquito aquatic habitats are rarely uniform in space or character. An orthogonal grid overlaid on satellite data of rice-land areas may fail to capture physical or man-made structures, i.e paddies, canals, berms at these habitats. Unlike an orthogonal grid, digitizing each habitat converts a polygon into a grid cell, which may conform to rice-land habitat boundaries. This research illustrates the application of a random sampling methodology, comparing an orthogonal and a digitized grid for assessment of rice land habitats.

**Methods:**

A land cover map was generated in Erdas *Imagine *V8.7^® ^using QuickBird data acquired July 2005, for three villages within the Mwea Rice Scheme, Kenya. An orthogonal grid was overlaid on the images. In the digitized dataset, each habitat was traced in Arc Info 9.1^®^. All habitats in each study site were stratified based on levels of rice stage

**Results:**

The orthogonal grid did not identify any habitat while the digitized grid identified every habitat by strata and study site. An analysis of variance test indicated the relative abundance of *An. arabiensis *at the three study sites to be significantly higher during the post-transplanting stage of the rice cycle.

**Conclusion:**

Regions of higher *Anopheles *abundance, based on digitized grid cell information probably reflect underlying differences in abundance of mosquito habitats in a rice land environment, which is where limited control resources could be concentrated to reduce vector abundance.

## Background

Rice land *Anopheles arabiensis *have adapted to take advantage of relatively ephemeral and irregular shaped aquatic habitats [1-5], where the remote measurement of larval abundance can be difficult due to their size and there temporal brevity. Overlaying a GIS grid on remotely sensed high resolution data can help organize and characterize mosquito larval habitats [6-11]. A grid is constructed by applying a mathematical algorithm in order to fit a continuous and bounded surface consisting of equidistant estimates of a quantity from a field sampled attribute [12]. GIS grid-based data files consist of columns and rows of uniform cells coded according to data values. Each grid cell within a matrix contains an attribute value as well as location coordinates. The spatial location of each cell is implicitly contained within the ordering of the matrix. As such aquatic habitats containing the same spatial attribute value are easily recognized.

However, due to asymmetrically shaped aquatic habitats in rice lands, an orthogonal grid may straddle habitat boundaries making statistical inferences of seasonal rice land *An. arabiensis *aquatic habitat data intolerable. Efficient handling of boundary conditions is a difficult problem in GIS grid-cell-based modeling [13]. Applying newer grid-based algorithms may spatially target rice land vector larval habitat abundance and distribution. For example, digitally tracing a rice land habitat in ArcInfo 9.1^® ^can generate polygons (grid cells) which may conform better to paddy and canal boundaries and help characterize mosquito larval habitats in relation to ecological attributes about a mosquito aquatic habitat. Digitized grid cell data using fine resolution satellite data may determine a reliable source of data sensitive to a number of rice land habitat features thought to be important for developing and implementing an integrated vector management (IVM) programme based on *An. arabiensis *larval productivity. QuickBird data can distinguish unambiguously between types of spatial objects and land uses solely in terms of detected spectral reflectance [14]. Therefore, the objective of this research was to compare an orthogonal grid and a digitized grid using QuickBird 0.61 m visible and near-infra red (NIR) data of three agro-village complexes within the Mwea Rice Scheme Kenya.

## Materials and methods

### Study area

The studies were conducted in Kangichiri, Kiuria and Rurumi villages within the Mwea rice scheme. The scheme is located 100 km north east of Nairobi. The scheme is situated on the foot hills of Mount Kenya at 112 km NE of Nairobi at 37°20'E and 0°, 41'S. Mwea is located at 1,159 m above sea level. It has a mean minimum and maximum temperature of 17°C and 26°C, respectively. The annual rainfall varies from a minimum of 356 mm to a maximum of 1,626 mm averaging 950 mm/year. Maximum precipitation takes place in April/May (long rains) and October/November (short rains). The average maximum temperatures are in the range of 16–26.5°C with relative humidity varying from 52–67%. Rurumi is located at the central-west region of the scheme and has 225 homesteads and 900 residents. Kangichiri and Kiuria are located in the north east of the scheme and have approximately 158 and 222 homesteads respectively with over 750 residents in each village.

### Larval sampling data

Paddies and canals located within a 1 km buffer in the Kangichiri, Kiuria and Rurumi study sites were identified and mapped using a CSI-Wireless differentially corrected global positioning systems (DGPS) Max receiver using an OmniStar L-Band satellite signal with a positional accuracy of less than 1 m (Advanced Computer Resources Corp (ACR), 100 Perimeter Road Nashua, NH, USA) [15]. Water bodies were inspected for mosquito larvae using standard dipping techniques with a 350 ml dipper to collect the mosquito larvae [16]. The number of dips per habitat was based on the size (0.3–1.0 ha) of the habitat and ranged between 15 and 25 dips. The larvae were placed in whirl pack bags and transported to the Mwea Research Station for further processing. Anopheline larvae were separated from culicine larvae, preserved in absolute ethanol and later identified morphologically to species [17]. The characteristics of each aquatic habitat were also recorded. A subset of the larvae of the *An. gambiae *complex were identified to sibling species using Polymerase Chain Reaction (PCR) technique [18].

### Stratifying mosquito habitats

Stratifying the rice land *An. arabiensis *aquatic habitats in the Kangichiri, Kiuria and Rurumi study sites involved assessing the level of rice development. The general hydrological duration of the rice cycle in the three study sites varies between 120–150 days. The cycle includes a field preparation phase comprising of flooding and ploughing stages prior to transplanting of rice seedlings, a post-transplanting stage with limited water, tillering, flowering/maturation and a post-harvest (fallow) season. Tillering extends from the appearance of the first tiller until the maximum tiller (5–9) number is reached. Stem elongation occurs and the tillers continue to increase in number and height, with increasing ground cover and canopy formation. The end of this stage marks the onset of flowering and maturation stage when plants stop growing and orient towards the development of the panicles and grains (water is drained), and in which plants senesce and their water content drops. The flowering/maturation phase lasts 20–30 days and includes the panicle initiation, booting, heading and flowering stages. Harvesting occurs 120–150 days post-transplanting when the grains fill, turn yellow and the plants senesce. After harvesting, the land is left fallow awaiting the next crop cycle.

### Habitat base-mapping

**Figure 1:**
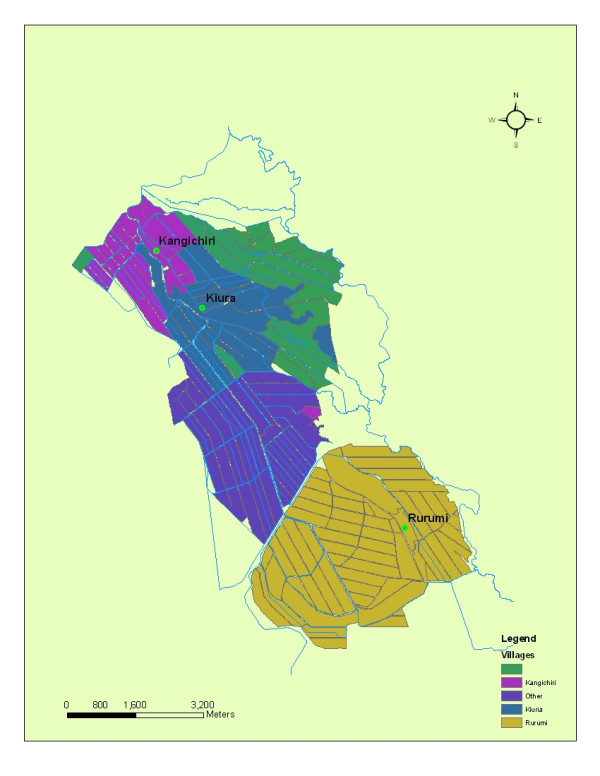
Base map of Kangichiri, Kuria and Rurumi study sites in the Mwea rice scheme Kenya.

Base maps for this study including major roads and hydrography were created using the DGPS data in ArcInfo 9.1^® ^(Earth Systems Research Institute Redlands, CA, USA, Figure 1). Each rice land *An. arabiensis *larval habitat with its associated land cover attributes from each study site were entered into a Vector Control Management System^® ^(VCMS, Advanced Computer Resources Corp (ACR), 100 Perimeter Road Nashua, NH, USA) database. VCMS supported the mobile field data acquisition in each village through a Microsoft PocketPC™. All two-way, remote synchronizing of data, geocoding, and spatial display were processed using the embedded GIS Interface Kit™ that was built using ESRI's MapObjects™ 2 technology. The VCMS database plotted and updated DGPS ground coordinates of aquatic larval habitats seasonal information and supported exporting data to a spatial format whereby any combination of rice land *An. arabiensis *larval habitats and supporting data in the Kangichiri, Kiuria and Rurumi study sites were exported in a GIS as a shape file format. This information was displayed onto a user-defined field base map.

### Remote sensing data

The QuickBird 0.61 m spatial resolution image encompassing the visible and NIR bands was acquired of the three study sites July16, 2005. Flooding in the Mwea rice scheme begins in late June in most rice cycles [19]. Mosquito larvae increase as soon as the paddies are flooded, rising to a peak when the rice plants are small, before declining when the rice plants cover the water surface [20-22]. Information from visible and NIR channels can distinguish between high and low mosquito producing rice fields [23]. QuickBird multispectral products provide 4 discrete non-overlapping spectral bands covering a range from 0.45 μm to 0.72 μm. QuickBird products are delivered as radiometrically corrected image pixels. The projection used for all of the spatial datasets is the UTM Zone 37S datum WGS-84 projection. After geographic registration alignment of the satellite's Universal Transverse Mercator (UTM) coordinates with known reference objects.

ArcInfo 9.1^® ^was used to integrate field data, such as DGPS coordinates and GIS data, with the Erdas *Imagine *from QuickBird data. The QuickBird 0.61 m visible and NIR data was classified using the Iterative Self-Organizing Data Analysis Technique (ISODATA) supervised routine in ERDAS *Imagine *V8.7^®^. A supervised classification can assign spectral signatures automatically generated by the ISODATA algorithm (24) for remote identification of mosquito aquatic habitats [9].

### Orthogonal grid

The grid cell size in the orthogonal grid was calculated by the mean size of the paddy in ArcInfo 9.1^®^. The area of the orthogonal grids that were overlaid on the QuickBird visible and NIR data were 64.3 m × 64.3 m in Kangichiri, 68.4 m × 68.4 m in Kiuria and 49.7 m × 49.7 m in Rurumi. Unique identifiers were placed in each grid cell unit. The grids extended out to a 1 km distance from the external boundary of each study site providing a 1 km radial area.

### Digitized grid

A digitized grid tracing each rice land *An. arabiensis *aquatic habitat was generated in Arc Info 9.1^® ^for the 3 study sites. Unique identifiers were placed in each grid cell (i.e., polygon). The grid extended out to a 1 km distance from the external boundary of each study site.

### Data analyses

The orthogonal grid and the digitized gridded image were 'screened' to determine fit and to provide an indication of the location of rice land *An. arabiensis *aquatic habitats. Due to the variation in the number of dips collected per habitat based on the size, we based our results on the number of larvae collected per dip. The entomological variable was total rice land anopheline *larvae*. An ANOVA test was performed to compare the differences in larval abundance between different paddy categories in each study site. An independent sample t-test was used to compare differences in larval abundance between paddies and canals as well as between vegetated and non-vegetated canals. Robust standard errors were used because data were collected at both the orthogonal grid cell and digitized grid cell level. An alpha level of 0.05 was used to indicate significance. All data management and calculations were performed using SAS 9.1.3^® ^(SAS inc. Carey, NC, USA).

## Results

**Figure 2:**
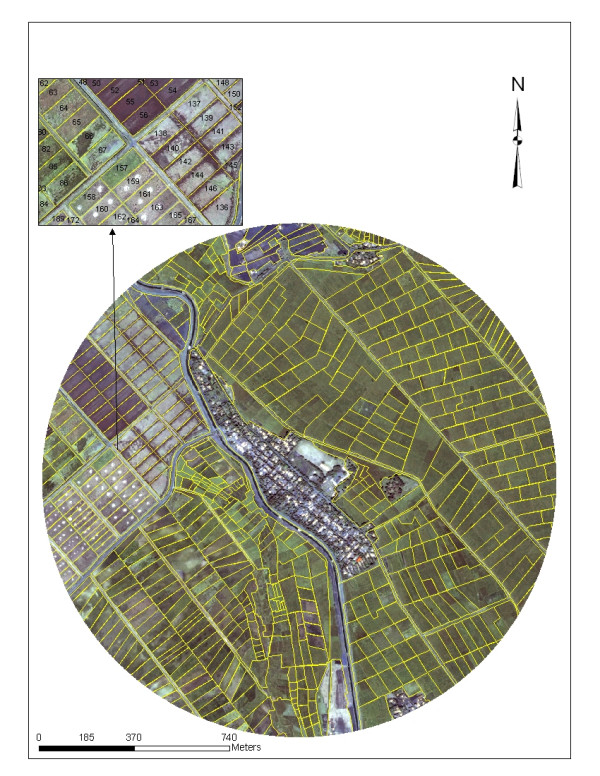
A digitized GIS grid overlaid on QuickBird 0.61 m image within 1 km buffer in Kangichiri agro-village, Mwea Rice Scheme, Kenya.

**Figure 3:**
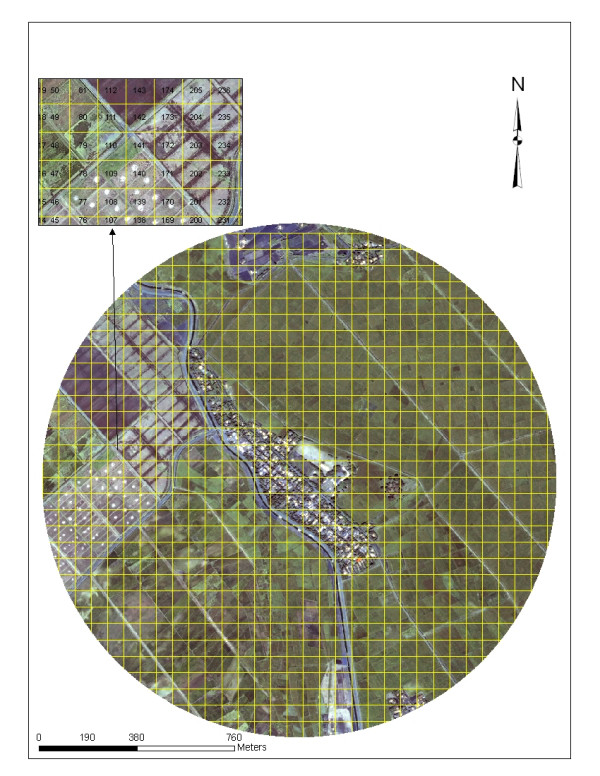
A 63.3 m × 63.3 m GIS overlaid on QuickBird 0.61 m image within 1 km buffer in Kangichiri agro-village, Mwea Rice Scheme, Kenya.

The digitized grid captured each rice land *An. arabiensis *aquatic habitat in the study sites (Figure 2) while the orthogonal grid was unable to identify any aquatic habitats in any of the three study sites (Figure 3).

The digitized grid cell data was used determine abundance of rice land *Anopheles *larvae in the paddy and canal habitats in each study site. The abundance of 1^st ^instar larvae/dip collected in Rurumi and Kangichiri study sites was 0.99 and 1.95, respectively and significantly lower than 4.81 in the Kiuria study site (F = 5.16, df 2, 751, p < 0.01). Similarly, the abundance of 2^nd ^instar larvae differed significantly among villages with that of 0.66 in the Rurumi study site being significantly lower than 1.09 or 2.11 in Kangichiri and Kiuria study sites, respectively (F = 3.79, df 2, 751, p < 0.05). The abundance of 3^rd ^and 4^th ^instar larvae as well as that of pupae did not differ significantly among villages (F = 1.64, 0.97 and 1.04, df 2, 75, p > 0.05). Table 1. shows the abundance of rice land *An. arabiensis *larvae/20 dips collected in the paddy and canal habitats at the 3 study sites. In the Kangichiri study site, the difference in the abundance of pupae and 1^st^, 2^nd ^and 3^rd ^instar larvae collected in paddy and canal habitats was not significant (p > 0.05) while that of 4^th ^instar larvae was significantly higher in the paddy habitats than in the canals (F = 5.19, df 1, 179, p < 0.05). In the Kiuria study site, significantly higher abundance of 3^rd ^instar larvae were collected in the canals (F = 4.68, df 1, 179, p < 0.05) while the other immature stages did not differ significantly between canal and paddy habitats. In the Rurumi study site, paddy habitats had significantly higher abundance of 1^st ^and 2^nd ^instar larvae compared with the canals (F = 5.60 and 3.94, df 1, 188, p < 0.05) but the other immature stages did not vary significantly between paddy and canal habitats.

**Table 1:** Abundance of *An. arabiensis *larvae in paddies and canals identified using digitized grid cell and field sampled data

Village	habitat type	No. of habitat	Proportion positive for *Anopheles *larvae	1^st ^instars	2^nd ^instars	3^rd ^instars	4^th ^instars	Pupae
Kangichiri	Paddy	160	57.10	1.64 ± 0.38	1.18 ± 0.25	0.24 ± 0.13	0.00 ± 0.00	0.40 ± 0.13
	Canal	135	42.90	2.28 ± 1.16	0.99 ± 0.25	0.17 ± 0.10	0.07 ± 0.03	0.17 ± 0.05
Kiuria	Paddy	122	62.80	5.50 ± 2.00	1.83 ± 0.59	0.14 ± 0.07	0.37 ± 0.35	0.27 ± 0.11
	Canal	69	37.20	3.66 ± 0.85	2.59 ± 0.85	0.40 ± 0.10	0.04 ± 0.03	0.19 ± 0.09
Rurumi	Paddy	106	68.60	1.42 ± 0.34	1.12 ± 0.45	0.08 ± 0.04	0.05 ± 0.03	0.16 ± 0.11
	Canal	98	31.40	0.59 ± 0.12	0.23 ± 0.07	0.11 ± 0.04	0.01 ± 0.01	0.04 ± 0.02

The distribution of larvae in rice fields at different stages of the rice cycle is shown in Table 2. Six stages of rice growth were identified in each of the three study sites by the digitized grid. An analysis of variance test indicated the relative abundance of immature stages of *An. arabiensis *at the three study sites to be significantly higher during the post-transplanting and the tillering stages of the rice growth (P < 0.05). In the other rice stages, the immature stages of *An. arabiensis *were either absent or occurring in low numbers.

**Table 2:** Immature stages of *An. arabiensis *sampled in paddies containing different stages of rice growth using digitized grid data

**Village**	**Paddy category**	**No. of Habitats**	**1**^st^**instars**	**2^nd ^instars**	**3^rd ^instars**	**4^th ^instars**	**Pupae**
Kangichiri	Ploughed	25	1.41	0.95	0.09	0.00	0.36
	Flooded	23	1.67	1.10	0.30	0.00	0.36
	Post transplanting	30	6.02	3.00	1.89	1.20	0.99
	Tillering	28	8.00	6.67	2.00	3.22	0.67
	Flowering/maturation	27	0.01	0.00	0.02	0.01	0.00
	Fallow	27	1.00	0.67	0.00	0.00	0.01

Kiuria	Ploughed	22	0.00	0.00	0.00	0.00	0.00
	Flooded	23	1.23	0.65	0.07	0.01	0.0
	Post transplanting	21	5.58	1.63	0.17	0.51	0.19
	Tillering	22	8.50	5.25	0.25	0.25	1.25
	Flowering/maturation	20	0.02	0.00	0.01	0.0	0.00
	Fallow	14	0.03	0.01	0.0	0.01	0.00

Rurumi	Ploughed	18	0.00	0.00	0.00	0.00	0.00
	Flooded	21	1.56	1.28	0.09	0.06	0.19
	Post transplanting	20	5.73	3.37	1.17	1.03	0.47
	Tillering	20	4.91	4.67	1.19	1.11	1.00
	Flowering/maturation	15	0.00	0.00	0.00	0.00	0.00
	Fallow	12	1.17	0.00	0.00	0.00	0.00

The relative abundance of rice land *An. arabiensis *larvae in vegetated and non-vegetated canal habitats identified by the digitized grid and the statistical comparisons of larval abundance between the two categories are represented in Tables 3 and 4. In the Kangichiri study site, the relative abundance of 1^st ^and 2^nd ^instar larvae was significantly higher in non-vegetated than vegetated canals while the differences in the other aquatic stages was not significant. In the Kiuria study site, the abundance of all the 4 larval instars of *An. arabiensis *was significantly higher in non-vegetated canals whereas in the Rurumi study site, the same trend was observed for the 2^nd ^and 3^rd ^instar larvae.

**Table 3:** Average number (± SE) of *An. arabiensis *larvae in vegetated and non-vegetated canals using the digitized grid data

VILLAGE	Vegetation	1^st ^instars	2^nd ^instars	3^rd ^instars	4^th ^instars	Pupae
Kangichiri	Present	1.20 ± 0.31	0.87 ± 0.21	0.00 ± 0.00	0.00 ± 0.00	0.00 ± 0.00
	Absent	24.50 ± 24.50	3.50 ± 3.50	0.18 ± 0.10	0.07 ± 0.03	0.17 ± 0.05
Kiuria	Present	0.00 ± 0.00	0.00 ± 0.00	0.00 ± 0.00	0.00 ± 0.00	0.00 ± 0.00
	Absent	3.83 ± 088	2.71 ± 0.89	0.72 ± 0.11	0.5 ± 0.03	0.20 ± 0.09
Rurumi	Present	0.4 ± 0.17	0.10 ± 0.10	0.00 ± 0.00	0.00 ± 0.00	0.00 ± 0.00
	Absent	0.64 ± 0.14	0.27 ± 0.08	0.14 ± 0.05	0.01 ± 0.01	0.05 ± 0.03

**Table 4:** Statistical values comparing the differences in *An. arabiensis *larval densities between the vegetated and non-vegetated canals

	Kangichiri			Kiuria			Rurumi		
	df	t	Sig.	df	t	Sig.	df	t	Sig.
1^st ^instars	134	22.22	0.00	68	0.86	0.03	97	0.87	0.35
2^nd ^instars	134	4.97	0.03	68	0.43	0.04	97	3.75	0.05
3^rd ^instars	134	0.15	0.70	68	0.68	0.04	97	0.02	0.04
4^th ^instars	134	0.23	0.64	68	0.08	0.06	97	0.01	0.93
Pupae	134	0.60	0.44	68	0.22	0.64	97	0.35	0.55

## Discussion

Results of this study demonstrated digitized grid cell data to be superior to orthogonal grid cell data for identification of rice land *Anopheles *aquatic habitats in the three study sites. Building a digitized grid geodatabase can identify relationships between rice land mosquito aquatic habitats, rice plant stage of development and agro-village-complex within the framework of relational database technology. By digitally tracing rice land *An. arabiensis *aquatic habitats using QuickBird visible and NIR data in GIS; we can generate polygons that approximate the curvature of the paddy and canal habitat boundaries. Field attribute tables can be associated to the polygons. Multiple data layers can be created using different coded values for various field attributes to the same digitized grid cell which can allow for multiple interactions between compatible ecological data bases enabling retrieval and transformation of seasonal larval habitat data efficiently regardless of spatial dimensionality of a rice land aquatic habitat.

Determining spatial heterogeneity in larval habitat distribution using a digitized grid cell database can have important operational significance because vector control operations can be designed to target zones where high larval densities occur. Treatments or habitat perturbations should be based on surveillance of larvae in the most productive areas of the agro-ecosystem and adjacent village [25]. The impact of larval control using new formulations of insecticides should be rigorously tested using a modified longitudinally designed study employing information from digitized grid cells. Laboratory studies should test *Bacillus thuringiensis ssp. israelensis *(Bti), *Bacillus sphaericus *(Neide) (Bsph) and Bti: Bsph ratios to determine lethal concentration parameters on all highly productive rice land *An. arabiensis *aquatic habitats in the three study sites. Confirmation of LC_50 _and LC_95 _values should be tested in each study site with two levels (high, low) of productivity. Overall product design goals may include: high efficacy (based on feeding behavior and susceptibility to bacteria toxins), minimal impact of ultraviolet radiation on efficacy, ease of use through conventional application equipment, and cost profile similar to other larvicides. Final candidate formulations should be evaluated in village-scale tests. Controlling a small proportion of productive rice land *An. arabiensis *aquatic habitats may yield significant reductions in a rice environment. For larval control, we assume that treatments applied to individual habitats are 100% effective in eliminating all immatures, i.e. treated habitats produce zero contribution to the total productivity.

In conclusion, the digitized grids identified and classified all rice land aquatic habitats examined in the three study sites by strata while the orthogonal grids did not identify any rice land *An. arabiensis *aquatic habitat in the study sites. The display of the rice land data in a digitized grid format clearly identifies highly productive rice land *An. arabiensis *aquatic habitats. Inventory of digitized grid cells regarding their larval productivity can provide critical information for characterizing rice land mosquito vector oviposition habitat selection and planning of IVM. Rice land mosquito surveillance systems based on an orthogonal grid may lack sensitivity for early warning as well as crucial larval abundance data for targeting effective prevention efforts.

## Authors' contributions

BGJ, helped conceive of the study and led the drafting of this manuscript; EJM, helped analyze the data; JF did the ArcInfo 9.1^® ^overlay operations and generated the gridded maps; JS supervised the field data collection and helped analyze the data; JG, and IK jointly conceived and designed the study; RJN is the principal investigator of the study. All authors interpreted the results and wrote the paper.
